# SR proteins and the nonsense-mediated decay mechanism are involved in human *GLB1 *gene alternative splicing

**DOI:** 10.1186/1756-0500-1-137

**Published:** 2008-12-29

**Authors:** Raül Santamaria, Lluïsa Vilageliu, Daniel Grinberg

**Affiliations:** 1 Departament de Genètica, Facultat de Biologia, Universitat de Barcelona, Barcelona, Spain; 2 CIBER de Enfermedades Raras (CIBERER), Barcelona, Spain; 3 Institut de Biomedicina de la Universitat de Barcelona (IBUB), Barcelona, Spain

## Abstract

**Background:**

The human *GLB1 *gene is known to give rise to two alternatively spliced mRNAs, which encode two different proteins: lysosomal β-galactosidase (β-gal) and elastin-binding protein (EBP). The β-gal transcript includes the 16 exons of the *GLB1 *gene. In the EBP transcript, exons 3, 4 and 6 are skipped, while exon 5 has a different reading frame. However, little is known on how this alternative splicing is regulated.

**Findings:**

Cycloheximide treatment of HeLa cells and human fibroblasts revealed the presence of new transcripts that are otherwise degraded by nonsense-mediated decay (NMD). A minigene carrying the exons involved in the alternative splicing of *GLB1 *was constructed. Improving the acceptor-site scores of exons 3 or 4 increased the relative inclusion of these exons, but did not stop them being skipped in some transcripts. Overexpression of different SR proteins altered the relative proportion of the different transcripts produced by the minigene, indicating a possible mechanism for the regulation of the alternative splicing of *GLB1*. Finally, a comparison of this gene among different species was performed.

**Conclusion:**

In the processing of the *GLB1 *RNA several transcripts are generated, but only those with a correct reading frame are not degraded. The differential inclusion/exclusion of exons could be partially explained by the relatively weak splice sites in the exons involved. Different SR proteins have an effect on the process of skipping of these exons, at least in the minigene conditions, indicating a possible mechanism for the regulation of the alternative splicing of the *GLB1 *gene.

## Background

The existence of splicing in eukaryotic RNAs has been known for more than 25 years [1,2]. Aternative splicing, which may affect 30 to 70% of the genes in higher eukaryotes [3,4], allows the generation of multiple transcripts by different mechanisms such as exon skipping/inclusion, alternative 5' or 3' splice sites, intron retention or mutually exclusive exons. Alternative exons often have suboptimal splice sites and/or suboptimal length when compared with constitutive exons. Besides the proteins that are present in constitutive splicing, a group of proteins that bind to pre-mRNA sequences are also necessary in alternative splicing [5]. The sequences where these proteins bind are called exonic or intronic enhancers (ESEs or ISEs) and silencers (ESSs or ISSs). Most of the ESEs are recognized by SR proteins. They constitute a family of proteins that has an RS domain (rich in arginines and serines) and 1 or 2 RRM domains (RNA-binding domains) and are essential, multifunctional splicing factors required both for spliceosome assembly and for alternative splicing [6]. On the other hand, the best characterized ESSs and ISSs are recognized by members of the heterogeneous nuclear ribonucleoprotein (hnRNP) family, which are highly abundant RNA-binding proteins that lack an RS domain [7]. It is known that these proteins have a dose-dependent effect and that the binding of SR and hnRNP proteins on the pre-mRNA allows the splicing machinery to act in a tissue or in a developmental stage specific manner [8].

Nonsense-mediated mRNA decay (NMD) is a well known mechanism that degrades mRNAs harbouring premature termination codons (PTCs), not only generated by *de novo *nonsense mutations but also of those naturally found in physiological transcripts [9]. Over recent years, the coupling between NMD and alternative splicing has become more evident [10].

The *GLB1 *gene (GenBank NM_000404.1), located on chromosome 3 at 3p21.33 and organized into 16 exons, is the gene coding for the β-galactosidase protein (E.C.3.2.1.23) [11]. This enzyme cleaves terminal β-galactoses from different substrates in the lysosome. A second transcript generated by alternative splicing was described [12], but it was not until 10 years later that the protein coded by this second transcript, the elastin-binding protein (EBP), was identified [13]. While the β-galactosidase cDNA is 2.5 kb long, the second transcript is only 2 kb, lacks exons 3, 4 and 6, and bears exon 5 in a different reading frame. The amount of the 2-kb transcript was also reported to be much lower (less than 1/10) than that of the 2.5-kb transcript [12].

Mutations in the *GLB1 *gene that result in the absence or reduced activity of the lysosomal enzyme β-galactosidase produce two different diseases: GM1-gangliosidosis (MIM# 230500) and Morquio B (MIM# 253010). A role of EBP in these diseases has also been suggested but is still under discussion [14,15].

Efforts have been made to establish the function and interactions of both proteins. In contrast, no studies have been reported on the way alternative splicing of the *GLB1 *gene is regulated. Here we present an attempt to establish the mechanisms underlying this alternative splicing.

## Methods

Vector construction, cell culture and transfection conditions, and molecular biology techniques are provided [see Additional file 1].

### Endogenous transcript analysis

**Figure 1:**
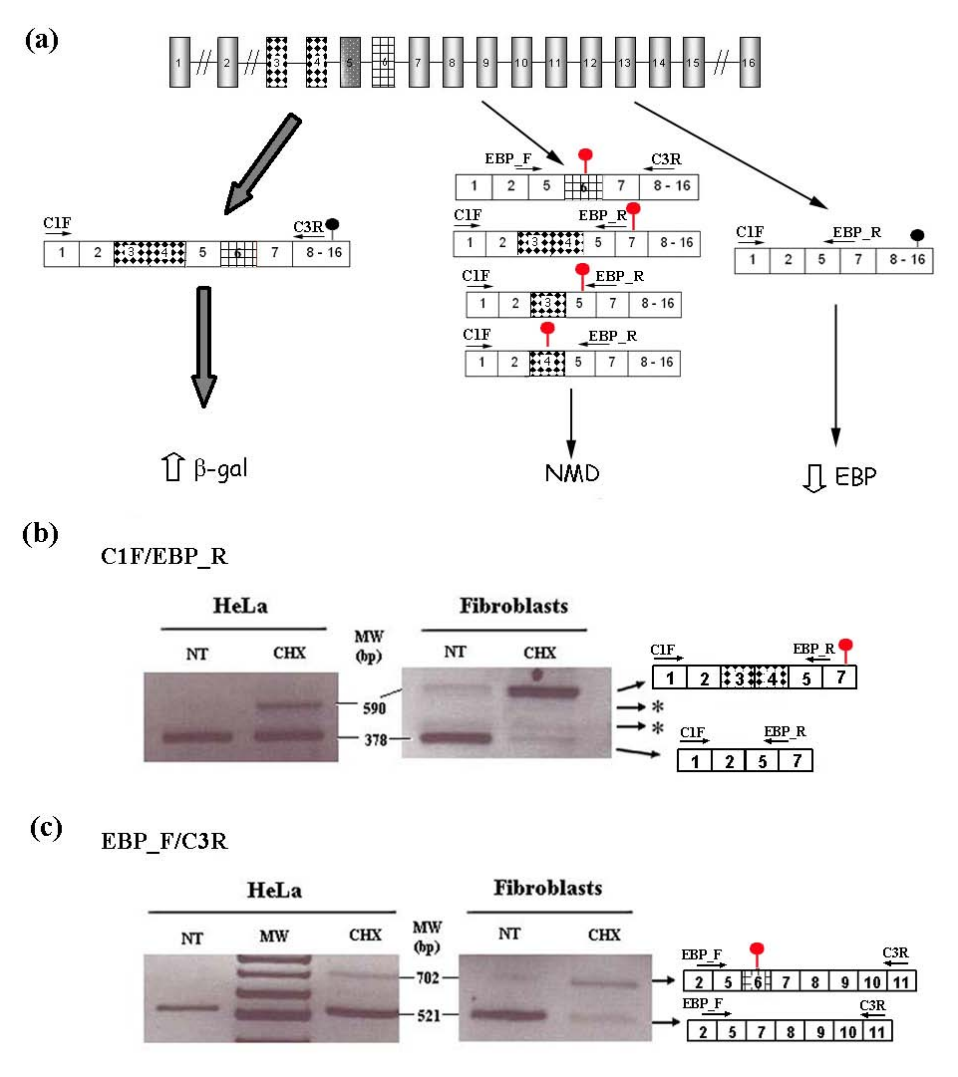
**Different transcripts of the *GLB1 *gene**. (a) Scheme of the *GLB1 *gene. Different possible transcripts are shown. The primers used to amplify these transcripts are indicated by horizontal arrows. Black circles are natural stop codons whereas red circles indicate PTCs. (b) &(c) RT-PCR on cDNA from cycloheximide-treated (CHX) or not treated (NT) HeLa cells and human fibroblasts. The transcripts corresponding to each band are outlined on the right. (b) PCR using C1F/EBPR as primers. (c) PCR using EBPF/C3R as primers. * indicates bands for possible transcripts including only exon 3 or only exon 4.

After treating cells with cycloheximide (CHX), besides the β-Gal and EBP transcripts, other transcripts bearing different combinations of exons 3, 4 and 6 were found. If these transcripts were generated, they would bear PTCs, because they would produce new reading frames (Figure 1a). RT-PCR using an EBP-specific primer (Table 1) revealed the presence of these intermediate transcripts in the CHX-treated cells, as shown in Figure 1b and 1c. The experiment was also performed on human control fibroblasts to rule out a possible specific effect of the transformed HeLa cell line. Again, bands corresponding to the intermediate transcripts were observed, although their relative abundance was different between the two cell types (Figure 1b, c). Two primers that should not anneal to any of the two natural transcripts were designed (2_4F and 4_6F). RT-PCR using the 2_4F/C3R or the 4_6F/C3R primers on cDNA from CHX-treated and untreated HeLa cells displayed a band only in the CHX-treated cells (data not shown).

**Table 1:** Primers and RT-PCR conditions

Primers employed in the RT-PCR				RT-PCRs performed	
Primer	Sequence (5'->3')	*GLB1*Exon^a^	Transcript specificity	Pairs of primers employed	AT (°C)

C1F	ACTGCAGAGCCGGGAGGCTGGT	1	EBP/β-Gal	C1F/EBPR	58

C3R	CCAAAGTGACCTTTCCATATG	11	EBP/β-Gal	EBPF/C3R	56.5

EBPF	GGCTGAACGCCATCCAGACATT	2–5	EBP	T7/SP6	55

EBPR	TGATGTTGCTGCCTGCACTG	7–5	EBP	T7/EBPR	56.5

2_4F	ACGCCATCCAGACGGAGGAT	2–4	NN	EBPF/SP6	58

4_6F	TCCTCCGACCCAGGTTGAA	4–6	NN	2_4F/C3R	59

T7	TAATACGACTCACTATAGGG	--	pcDNA3	2_4F/SP6	58.3

SP6	GATTTAGGTGACACTATAG	--	pcDNA3	4_6F/C3R	59

C1Fm	GAGACCCCATCGTGGCGCGA	1	m EBP/β-Gal	C1Fm/C1Rm	59

C1Rm	CTCTAGTAGCCAAGCAGGTAAGC	4	m β-Gal	EBPFm/EBPRm	58

EBPFm	GGGCTGAATGCTATCCAGATAC	2–5	m EBP		

EBPRm	TGTGATATTGTTGCCTGCACGGT	7–5	m EBP		

AT: annealing temperature; NN: not naturally ocurring; m:mouse.

^a^*GLB1 *exon or exons that are complementary to the indicated primers

### Minigene transcript analysis

**Figure 2:**
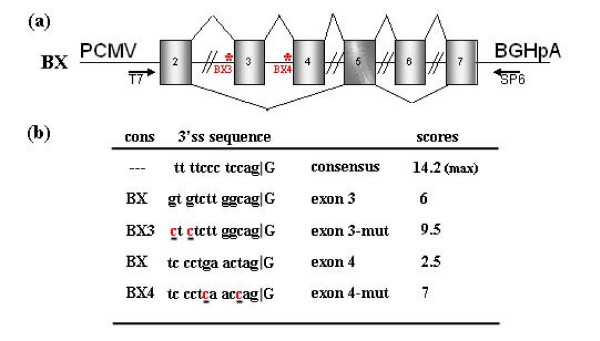
**BX minigene: mutated splice sites and scores**. (a) Scheme of the BX minigene construct. Exons: grey boxes. Introns: horizontal lines. Two lines cutting introns indicate that the whole intron was not cloned. The naturally occurring β-Gal (above the exons) and EBP (below the exons) splicing pathways are indicated. * (in red) indicates the location of the changes introduced by site-directed mutagenesis in BX3 and BX4 constructs. Arrows indicate the location of T7 and SP6 primers. PCMV: CMV promotor; BGHpA: BGH polyadenylation site. (b) 3'ss wild-type and mutated sequences and calculated splicing scores. Changes introduced by mutagenesis are underlined. cons: plasmid construct.

In order to study *GLB1 *alternative splicing, a minigene (BX) including the alternatively spliced region of the gene (exons 2, 3, 4, 5, 6 and 7) was constructed (Figure 2a). The BX minigene was made so that the different combination of exons generated transcripts without PTCs. This allowed us to completely discard the effect of NMD as the cause of the observed pattern of bands.

To determine whether some of the missing intronic sequences were essential for the correct splicing of any of the cloned exons, the BX construct was transfected in HeLa cells and RT-PCR was carried out using plasmid-specific primers (T7/SP6). A single band was obtained (data not shown). Sequencing of this PCR product showed that it corresponded to a transcript that included the 6 cloned exons, indicating that the intron regions present in the BX construct bore the necessary *cis *sequences for correct splicing. If alternative splicing giving rise to the EBP transcript was taking place, it was at a very low level (as in the endogenous splicing), since no band corresponding to this transcript was detected.

**Figure 3:**
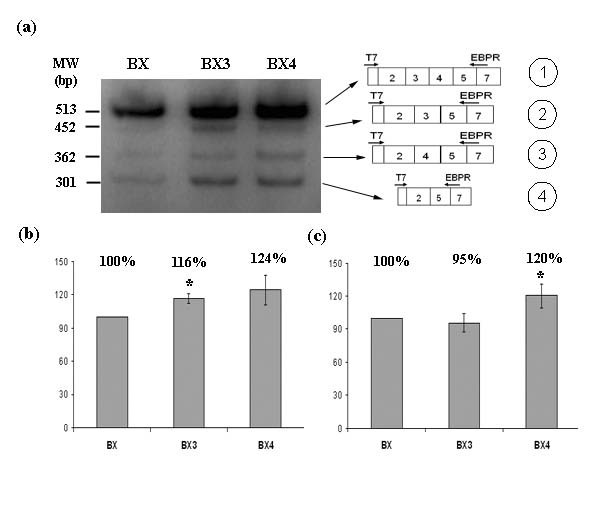
**Semiquantitative RT-PCR on cDNA from transfected HeLa cells with plasmids BX, BX3 and BX4**. (a) Semiquantitative RT-PCR using T7/EBPR primers (left) and scheme of the transcript corresponding to each band is shown (right). (b) Percentage of transcripts including exon 3 versus transcripts lacking it, i.e. (1+2)/(3+4). (c) Percentage of transcripts including exon 4 versus transcripts lacking it, i.e. (1+3)/(2+4). Each value is the mean ± S.E. of two independent experiments, each of which was replicated three times. * when p < 0.05.

In order to check whether EBP-specific splicing occurred at all, RT-PCR was performed with EBP-specific primers. Both T7/EBPR and EBPF/SP6 pairs of primers produced a PCR band corresponding to the EBP transcript. However, the band corresponding to the EBP transcript was a minor one and extra bands, corresponding to other intermediate transcripts, were also observed. The BX lane in Figure 3a shows the results for the T7/EBPR pair of primers. Similar results were obtained for EBPF/SP6 (not shown).

### Effect of exon 3 and exon 4 acceptor sites on alternative splicing

*In silico *analysis of the 3' splice sites (3'ss) of the *GLB1 *gene revealed a low score of 2.5 for the site flanking exon 4, a relatively low score of 6 for the site adjacent to exon 3 and a high score of 10 for exon 6 acceptor site, compared to the average score of constitutive exons (7.9). To check whether the skipping of exons 3 and 4 in the alternative splicing was due to the weak acceptor sites, 2 positions on each 3'ss were mutagenized in the BX minigene, generating the BX3 and BX4 constructs. These changes increased the 3'ss scores on exons 3 and 4, from 6 to 9.5 and from 2.5 to 7, respectively (Figure 2a, b). Analysis of the transcripts generated by these plasmids showed an increase of the transcript including only exon 3 (BX3 lane) and of the transcript including only exon 4 (BX4 lane), although the transcripts lacking both exons were also observed (Figure 3a). Quantification of the transcripts bearing exon 3 (or exon 4) versus transcripts lacking exon 3 (or exon 4), showed small but significant increases. As expected, plasmid BX3 produced proportionally more transcripts bearing exon 3 than BX (116%, Figure 3b), and plasmid BX4 produced more bearing exon 4 (120%, Figure 3c). It should be noted that plasmid BX4 also produced more transcripts bearing exon 3 than BX (124%), but this was not significant. Nevertheless, the fact that some transcripts with skipped exons 3 and/or 4 were still produced, suggests that the weak 3' splice sites are not sufficient to explain why these exons might be skipped.

### Effect of over-expression of SR proteins on alternative splicing

**Figure 4:**
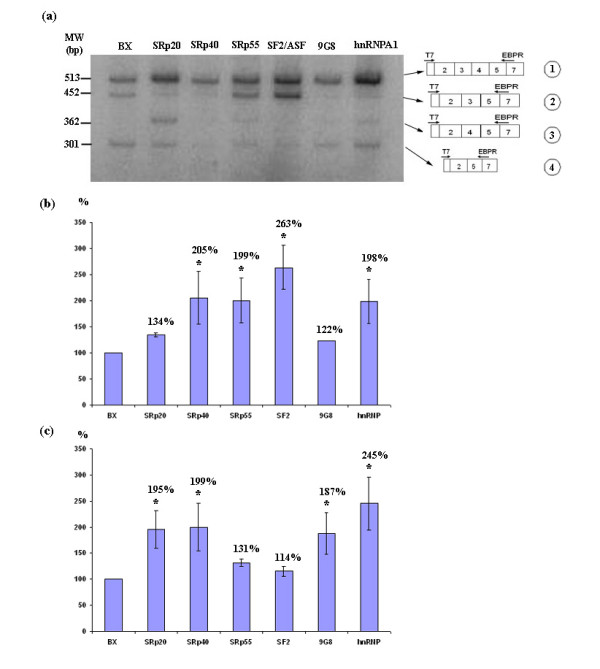
**RT-PCR using primers T7/EBPR after cotransfection with the BX and SR plasmids**. (a) Semiquantitative RT-PCR, using primers T7/EBPR, on cDNA from HeLa cells cotransfected with the BX plasmid together with a plasmid expressing one of the following proteins: SF2/ASF, SRp20, SRp40, SRp55, 9G8 and hnRNPA1. The BX lane is the control. The transcripts corresponding to each band are drawn on the right. (b) &(c) Quantification of radio-labelled RT-PCR for the different transcripts (b for transcripts including exon 3 versus transcripts lacking it, i.e. (1+2)/(3+4) and c for transcripts including exon 4 versus transcripts lacking it, i.e. (1+3)/(2+4)), considering BX transfection as 100% in each case. Each value is the mean ± S.E. of two independent experiments, each of which was replicated three times. * when p < 0.05.

To assess the effect of the SR and hnRNP A/B proteins on *GLB1 *alternative splicing, HeLa cells were cotransfected with the BX plasmid together with a plasmid expressing one of the following proteins: SF2/ASF, SRp20, SRp40, SRp55, 9G8 or hnRNPA1. The analysis was focused on the inclusion/exclusion of exons 3 and 4. The different transcripts detected by RT-PCR using primers T7/EBP_R were quantified. Although none of these proteins could completely restore the inclusion of one of the exons (3 or 4), important variations in the relative proportion between the different transcripts were detected (Figure 4a–c). The cotransfection of the BX plasmid plus an empty pcDNA3 plasmid was used as a control (BX lane). Quantification of the bands showed that the SR proteins reduced the relative amount of the EBP transcript, compared to the transcript that includes all the exons. Additionally, most of the SR proteins increased the amount of transcripts including exons 3 or 4 (Figure 4b, c). This indicates a function for SR proteins favouring the inclusion of these exons, although some seem to favour both exons (SRp40) and others only exon 3 (SRp55 and SF2/ASF) or exon 4 (SRp20 and 9G8). On the other hand, hnRNPA1, which has been reported to inhibit splicing [7], here seems to favour the inclusion of both exons too. However, an *in vitro *splicing enhancement effect of hnRNPA1 has also been reported [16]. Previous studies have found a close similarity between the RRMs of proteins 9G8 and SRp20, which can recognize some identical sequences [17], and moreover, it has also been reported that 9G8 can participate in splicing regulation as a functional SRp20 homologue in certain conditions [18]. In our study, both proteins produced the same change of pattern (that is, favour the inclusion of exon 4), consistent with these previous results.

**Figure 5:**
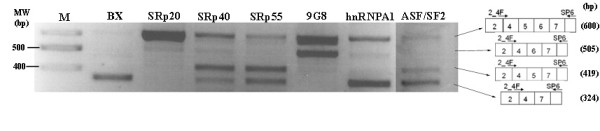
**RT-PCR using primers 2_4F/SP6 after cotransfection with the BX and SR plasmids**. RT-PCR, using primers 2_4F and SP6, on cDNA from HeLa cells cotransfected with the BX plasmid together with a plasmid expressing one of the following proteins: ASF/SF2, SRp20, SRp40, SRp55, 9G8 and hnRNPA1. The transcripts corresponding to each band are drawn on the right.

To analyse whether the effect of the SR proteins was specific for exons 3 and 4 or if it was a general phenomenon, a similar experiment was performed using the 2_4F/SP6 primers to measure the effect on the inclusion/exclusion of exons 5 and 6 (Figure 5). The results showed again that the SR proteins had a general effect of favouring the inclusion of exons 5 and 6. The experiment was repeated three times with similar results.

The differences in the pattern observed for each protein may indicate their different roles in the constitutive and alternative splicing of these exons. However, it should be taken into account that the over-expression of the SR proteins in the minigene experiments, which is far from being a physiological situation, could alter the interactions between the over-expressed and endogenous SR proteins. Moreover, the characteristics of the minigene, such as the type of promoter or the lack of some of the intronic sequences, should also be considered.

### *GLB1 *alternative splicing in other species

It had already been described that the EBP could not be generated in mouse (*Mus musculus*) through the alternative splicing of the *GLB1 *gene, because the translation of exon 5 in mouse is only possible in the reading frame that codes for lysosomal β-galactosidase [19]. Analysis of the *GLB1 *exon 5 sequences in different species showed that this also happens in rat (*Rattus norvegicus*). However, in other mammals such as dog (*Canis familiaris*), cat (*Felis catus*), crab-eating macaque (*Macaca fascicularis*) or rabbit (*Oryctolagus cuniculus*) and also in birds such as the chicken (*Gallus gallus*), both reading frames are possible. Therefore, in all the species analyzed *in silico*, except for rat and mouse, a reading frame where exons 3, 4 and 6 are skipped is possible.

To see if the EBP alternative splicing (i.e. exclusion of exons 3, 4 and 6) occurred in mice, RAW264.7 cells were cultured in the presence and absence of CHX, and β-Gal- and EBP-like-specific RT-PCR were carried out. The C1Fm/C1Rm primers were used for β-Gal and the C1Fm/EBPRm primers for EBP-like (Table 1). The EBP-like transcript was detected only in the CHX-treated cells (data not shown).

The existence of this EBP-like transcript suggests that its absence in rodents (*Murinae*) could be explained by the presence of the PTC (a change that is synonymous in the β-gal frame) and the corresponding degradation by NMD. The question of which is the gene coding for the mouse EBP protein, if it exists, or which protein performs a similar function, remains open.

## Competing interests

The authors declare that they have no competing interests.

## Authors' contributions

RS performed the experiments. LV and DG designed the experiments and supervised the work. RS, LV and DG wrote the paper. All authors read and approved the final manuscript.

## Supplementary Material

Additional file 1 **Methods.** The file contains a detail description on vector construction, cell culture and transfection conditions, and molecular biology techniques used in this work. Click here for file
