# Supplementary material for: SR proteins and the nonsense-mediated decay mechanism are involved in human GLB1 gene alternative splicing
Source: BMC Res Notes. 2008 Dec 29;1:137. doi: 10.1186/1756-0500-1-137 (PMC2631023; doi:10.1186/1756-0500-1-137)
Supplement: Additional file 1 — Methods. The file contains a detail description on vector construction, cell culture and transfection conditions, and molecular biology techniques used in this work. [file 1756-0500-1-137-S1.doc]

**Methods**

**Vector construction**

*In vitro* studies of *GLB1* alternative splicing were performed using a minigene construct carrying exons 2, 3, 4, 5, 6 and 7. Since the introns involved are very long (more than 15 kb), only the exons and their flanking sequences (about 200 bp on each side) were cloned in a pcDNA3.1 plasmid. Fragments were PCR-amplified with primers including terminal restriction sites useful for cloning (primer sequences are available on demand). The pCGT7 plasmids bearing cDNAs encoding SR proteins (SF2/ASF, SRp20, SRp40, SRp55, 9G8) and hnRNPA1 were kindly provided by A. R. Kornblihtt and were described elsewhere [1].

**Cell culture and transfection**

HeLa cells, human fibroblasts and RAW264.7 mouse cells were cultured in the presence of DMEM medium (GIBCO, BRL Grand Island, NY, USA) with 10% foetal bovine serum (GIBCO, BRL Grand Island, NY, USA) and antibiotics, at 37ºC and 5% CO_2_. CHX treatment was performed at 500 µg/ml. When cells were 90% confluent, CHX was added to the medium for 4 hours and then RNA was isolated.

For transfection, cells were plated at 50% of confluency in 6-well culture plates and 24 hours later, at 90% of confluency, 500 ng of the corresponding plasmid were mixed with 4µl of *Lipofectamine^TM^ 2000 Reagent* (Invitrogen, Carlsbad, CA), according to the manufacturer’s recommendations. In cotransfections, 500 ng of each plasmid were used. As a control, the minigene plasmid (BX) was mixed with 500 ng of an empty pcDNA3.1 vector. Cells were collected 48 hours after transfection. RNA isolation, cDNA synthesis, RT-PCR fragment purification and sequencing were performed as previously described [2]. Expression of SR proteins was always checked by RT-PCR of the specific transcript.

**PCR amplification**

Endogenous and minigene transcripts were analyzed by conventional and semiquantitative RT-PCR. PCRs were performed using 1 U of Taq DNA Polymerase (Promega, Madison, WI, USA), 10 pmols of each primer (see Table 1), 2.5 mM MgCl_2_, 200 µM dNTPs, 0.1 µl ^32^α-P dATP (10µCi/µl) (in semiquantitative PCRs) and 5-10 ng of cDNA in the recommended buffer. PCR conditions were as follows: 4 min of denaturation at 95ºC, 25 (or 35 in the non-quantitative PCRs) cycles of denaturation at 94ºC for 30s, annealing for 30s at the temperature indicated in Table 1 and extension for 30s at 72ºC. For the amplification of minigene transcripts, a transcript-specific primer and a plasmid-specific primer (T7 or SP6) were used to avoid amplification of endogenous transcripts.

Transcript specificity was checked for the EBP primers. EBPF primer was complementary to exon 2 and exon 5 sequences (at its 5’ end to exon 2 and at its 3’ end to exon 5). EBPR primer was complementary to exons 7 and 5 (at its 5’ end to exon 7 and at its 3’ end to exon 5). As a control of transcript specificity, “scrambled” primers were used. The scrambled EBPF primer was synthesized with the same exon 5 sequence as the EBPF primer, but with the nucleotides corresponding to exon 2 scrambled. The same was performed for the scrambled EBPR primer, where nucleotides corresponding to exon 7 were scrambled. The absence of any amplification in the PCRs performed with scrambled primers confirmed the specificity of the EBP primers.

In order to quantify the resulting semiquantitative PCR products, 6 µl of each sample were loaded on a non-denaturing 5% polyacrylamide gel and run for 1h 30min at 14 mA. Gels were then dried and visualized in a *Molecular Imager FX* (Biorad). Quantification was performed using the *Quantity One®* (Biorad) software.

**Site-directed mutagenesis**

Changes at the splicing acceptor sites of exons 3 and 4 were carried out by site-directed mutagenesis on the minigene construct, using the *QuickChange^TM^ Site-Directed Mutagenesis XL kit* (Stratagene, La Jolla, CA) following the manufacturer’s instructions. In particular, two nucleotide changes were introduced on each 3’ss. Changes introduced in the acceptor site of exon 3 were: c.246-10G>C and c.246-12G>C (BX3 construct); and in the acceptor site of exon 4: c.397-3T>C and c.397-7G>C (BX4 construct).

**Informatic support and statistical analysis**

The acceptor-site scores were analyzed using the software developed by Zhang and Yada at [http://rulai.cshl.edu/new_alt_exon_db2/HTML/score.html](http://rulai.cshl.edu/new_alt_exon_db2/ HTML/score.html).

In semiquantitative PCRs, each transfection experiment was performed at least twice. At least 3 PCRs of each transfection were performed to quantify the relative proportion of each transcript. The Mann-Whitney U-test was used to analyze significant differences in band intensities between those corresponding to control and treated cells.

**References (Methods)**

1. Caceres JF, Misteli T, Screaton GR, Spector DL, Krainer AR: **Role of the modular domains of SR proteins in subnuclear localization and alternative splicing specificity**. *J Cell Biol* 1997, **138**(2):225-238.

2. Santamaria R, Chabas A, Coll MJ, Miranda CS, Vilageliu L, Grinberg D: **Twenty-one novel mutations in the GLB1 gene identified in a large group of GM1-gangliosidosis and Morquio B patients: possible common origin for the prevalent p.R59H mutation among gypsies**. *Hum Mutat* 2006, **27**(10):1060.
